# Diversity and life-cycle analysis of Pacific Ocean zooplankton by videomicroscopy and DNA barcoding: Hydrozoa

**DOI:** 10.1371/journal.pone.0218848

**Published:** 2019-10-25

**Authors:** Peter J. Bryant, Timothy E. Arehart

**Affiliations:** 1 Department of Developmental and Cell Biology, University of California, Irvine, Irvine, CA, United States of America; 2 Crystal Cove Conservancy, Newport Coast, CA, United States of America; Tierarztliche Hochschule Hannover, GERMANY

## Abstract

Most, but not all cnidarian species in the class Hydrozoa have a life cycle in which a colonial, asexually reproducing hydroid phase alternates with a free-swimming, sexually reproducing medusa phase. They are not well known, in part because many of them are microscopic, at least in the medusa phase. Matching the two phases has previously required rearing of the organism from one phase to another, which has not often been possible. Here we show that DNA barcoding makes it possible to easily link life-cycle phases without the need for laboratory rearing. Hydrozoan medusae were collected by zooplankton tows in Newport Bay and the Pacific Ocean near Newport Beach, California, and hydroid colonies were collected from solid substrates in the same areas. Specimens were documented by videomicroscopy, preserved in ethanol, and sent to the Canadian Centre for DNA Barcoding at the University of Guelph, Ontario, Canada for sequencing of the COI DNA barcode. In the order Anthoathecata (athecate hydroids), DNA barcoding allowed for the discrimination between the medusae of eight putative species of *Bougainvillia*, and the hydroid stages were documented for two of these. The medusae of three putative species of *Amphinema* were identified, and the hydroid stages were identified for two of them. DNA barcodes were obtained from medusae of one species of *Cladonema*, one adult of the by-the wind Sailor, *Velella velella*, five putative species of *Corymorpha* with the matching hydroid phase for one; and *Coryne eximia*, *Turritopsis dohrnii* and *Turritopsis nutricula* with the corresponding hydroid phases. The actinula larvae and hydroid for the pink-hearted hydroid *Ectopleura crocea* were identified and linked by DNA barcoding. In the order Leptothecata (thecate hydroids) medusae were identified for *Clytia elsaeoswaldae*, *Clytia gracilis* and *Clytia sp*. *701 AC* and matched with the hydroid phases for the latter two species. Medusae were matched with the hydroid phases for two species of *Obelia* (including *O*. *dichotoma*) and *Eucheilota bakeri*. *Obelia geniculata* was collected as a single hydroid. DNA barcodes were obtained for hydroids of *Orthopyxis everta* and three other species of *Orthopyxis*. One member of the family Solmarisidae, representing the order Narcomedusae, and one member (*Liriope tetraphylla*) of the order Trachymedusae were recognized as medusae. The results show the utility of DNA barcoding for matching life-cycle stages as well as for documenting the diversity of this class of organisms.

## Introduction

Cnidarians are divided into five classes [1]: Hydrozoa (Usually small, with either or both hydroid and medusoid phases); Anthozoa (Sea Anemones); Scyphozoa (Jellyfish); Staurozoa (Stalked Jellyfish) and Cubozoa (Box Jellyfish). The Anthozoa and Scyphozoa are well known, but the class Hydrozoa is less well known, in large part because many of the species are microscopic. Members of the class Staurozoa are interpreted as attached medusoid forms, and are usually found in cold waters [2]. The class Cubozoa [3] has been documented off Southern California by a single species collection in San Diego County [4]. An additional class Endocnidozoa, consisting of small parasitic forms in the subclasses Polypodiozoa-[5] and Myxozoa [6] has recently been proposed.

Many species of the class Hydrozoa have a life cycle in which a sexually reproducing, free-swimming medusa phase alternates with an asexually reproducing sessile hydroid stage. The latter often exists as a colony, which can be either male or female, giving rise by asexual reproduction to male or female medusae, respectively. Both body forms show a basic radial symmetry, with a mouth surrounded by tentacles leading into the body cavity where digestion occurs. The hydroid phase often develops a creeping stolon with attached branches that carry the tentacles and mouth distally; in these cases the radial symmetry is clearest only at the distal tips. Although members of the class Hydrozoa are fairly inconspicuous, at both phases of the life cycle they are both predators and prey in marine food chains, so they are important in ocean ecology. In some species, either the medusa or the hydroid phase is missing. A related freshwater species (Hydra) in which the medusa phase is missing, is an important model system for studies in developmental biology.

We have collected specimens of hydrozoans from our local waters, and used DNA barcoding to match life-cycle stages and to document the level of biodiversity within this group.

## Methods

Zooplankton collections were made from 19 sites (Table 1) under Scientific Collecting Permit SC-12162 from the California Department of Fish and Wildlife.

**Table 1:** Collection locations.

Locality	Latitude	Longitude
Balboa Island Coral Street	33D 36'N	117D 53'W
Balboa Pavilion Dock	33D 36'N	117D 54'W
Bayside Marina	33D 37'N	117D 54'W
Back Bay Science Center Dock	33D 37'N	117D 53'W
Cabrillo Marine Aquarium	33D 43'N	118D 17'W
Crew dock, BBSC	33D 37'N	117D 53'W
Delhi Channel	33D 38'N	117D 53'W
Lido Island (Via lido and Genoa)	33D 36'N	117D 55'W
Mussel Rocks, Crystal Cove State Park	33D 34'N	117D 50'W
Newport Harbor	33D 36'N	117D 53' W
Newport Harbor Entrance	33D 35'N	117D 53'W
Newport Pier	33D 36'N	117D 55'W
Ocean off Crystal Cove State Park	33D 33'N	117D 51'W
Ocean off Dana Point	33D 27'N	117D 42'W
Ocean off Newport Beach	33D 34'N	117D 53'W
Off Newport Aquatic Center	33D 37'N	117D 53'W
Under Pacific Coast Highway Bridge	33D 37'N	117D 54'W
Reef Point, Crystal Cove State Park	33D 34'N	117D 50'W
San Clemente Beach	33D 25'N	117D 37'W

Shore-based collections were made with a 150 μm mesh net (aperture 30 cm) attached to a rope, with a 50 ml collection tube at the base. They were made from a public dock using repeated horizontal sweeps near the surface and diagonal sweeps down to about 15 ft. depth. About 5–10 sweeps of a total of about 100 feet usually yielded sufficient specimens, but no attempt was made to monitor collections quantitatively. Collections were made and analyzed with the assistance of Undergraduate students Taylor Sais, Alicia Navarro, Debbie Chung and Lesly Ortiz.

Ocean collections were made with a 250 μm mesh net attached to a 100 ft. rope. The net (aperture 30 cm) was towed behind the vessel, just below the surface, for a period of 7 minutes at the slowest possible speed. Deployment and retrieval extended the total tow period to 10 minutes.

One specimen of *Cladonema californica* was found in tanks at Orange Coast College, Orange County, CA on 7/19/2018. It probably arrived in a shipment of sea urchins collected in Long Beach, CA.

Collections were brought to the laboratory at the University of California, Irvine and examined under a dissecting microscope with lateral light and a dark background. Each specimen of interest was removed using a Pasteur Pipette, transferred to a depression slide, and recorded by video microscopy using a Zeiss microscope with a dark-field condenser, fitted with a phototube attached to a Nikon D5100 single-lens reflex camera. The most informative frames were taken from the videos and used in the figures for this paper. Each specimen was preserved in 90% ethanol in a well of a 96-well microplate. Filled plates were sent to the Canadian Centre for DNA Barcoding at the University of Guelph for sequencing of the standard 648-bp “DNA barcode” [7] in the COI mitochondrial gene, using the following primers: (LCO1490 GGTCAACAAATCATAAAGATATTGG; HCO2198 TAAACTTCAGGGTGACCAAAAAATCA). This usually produced a DNA barcode of 658 nucleotides, and only those containing >/ = 650 nucleotides were included in the sequence analysis. Species that could not be identified morphologically were assigned operational taxonomic names of the form nPJB, and groups of specimens with identical or almost identical DNA barcodes were assigned BIN numbers. The DNA sequences are in the public domain at the Canadian Centre for DNA Barcoding.

Hydroid phase specimens were obtained by removing bunches of seaweed from docks in Newport Bay, bringing them to the laboratory and examining them under the dissecting microscope. This provided hydroid stages for many but not all of the medusae investigated, presumably because hydroids of some species live on the seabed or in other locations that are difficult to access for collections. Hydroid specimens were recorded and processed in the same manner as medusae.

## Results

**Fig 1:**
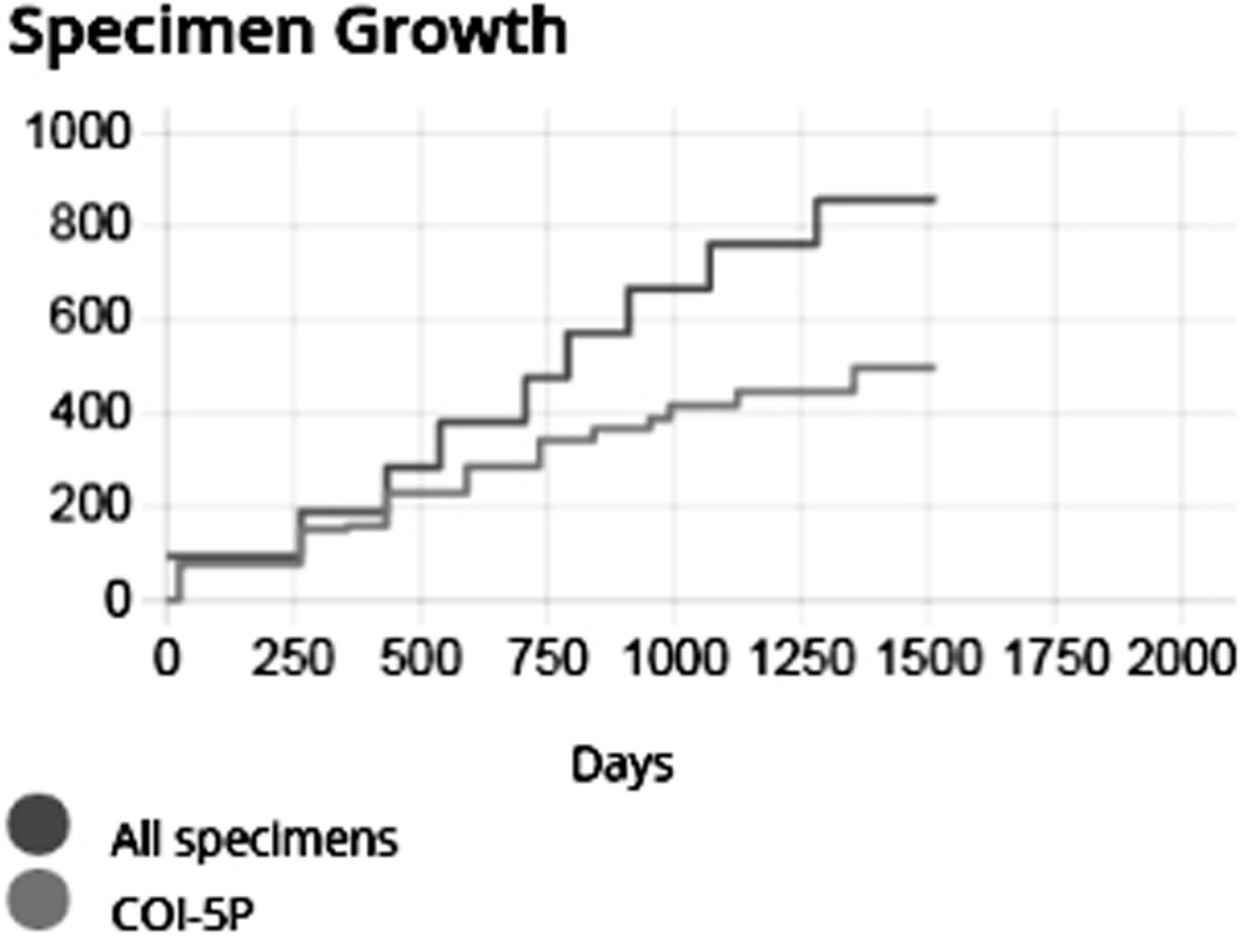
Specimen growth curve for the collection described in this paper.

From 843 Specimens, 497 sequences were obtained falling into 97 BINs, containing 39 recognized species. The specimen growth curve (Fig 1) shows that this collection is approaching saturation, but has not yet reached it.

Here we follow the taxonomy from Daly et al. [1]: * Representatives found in the current collection.

Class Hydrozoa; Subclass Hydroidolina

* Order Anthoathecata (with athecate hydroids)

* Order Leptothecata (with thecate hydroids)

Order Siphonophorae

Subclass Trachylina

Order Actinulida

Order Limnomedusae

* Order Narcomedusae

* Order Trachymedusae

### Order Anthoathecata: With Athecate Hydroids

The hydroid stalk is athecate (not surrounded by a sheath).

#### Family Bougainvilliidae

Medusae of local species in this family are all microscopic and can be distinguished by the number of tentacle bases and the number of tentacles attached to each base [8]. The digestive system hangs from the center of the umbrella, leading to the manubrium with the mouth at its tip. Most species in the literature also have four small tentacles (oral tentacles) surrounding the mouth, and these tentacles are usually dichotomously branched. The gonads, producing sperm in the males and eggs in the females, are attached to the sides of the manubrium.

The hydroid stock is athecate, the hydranths having a conical proboscis and a single whorl of filiform tentacles; the medusa buds are borne below the hydranths, on their pedicels or on the stems. The hydroids in this family are very difficult to identify because they show much less obvious morphological diversity than the corresponding medusa phases.

**Fig 2:**
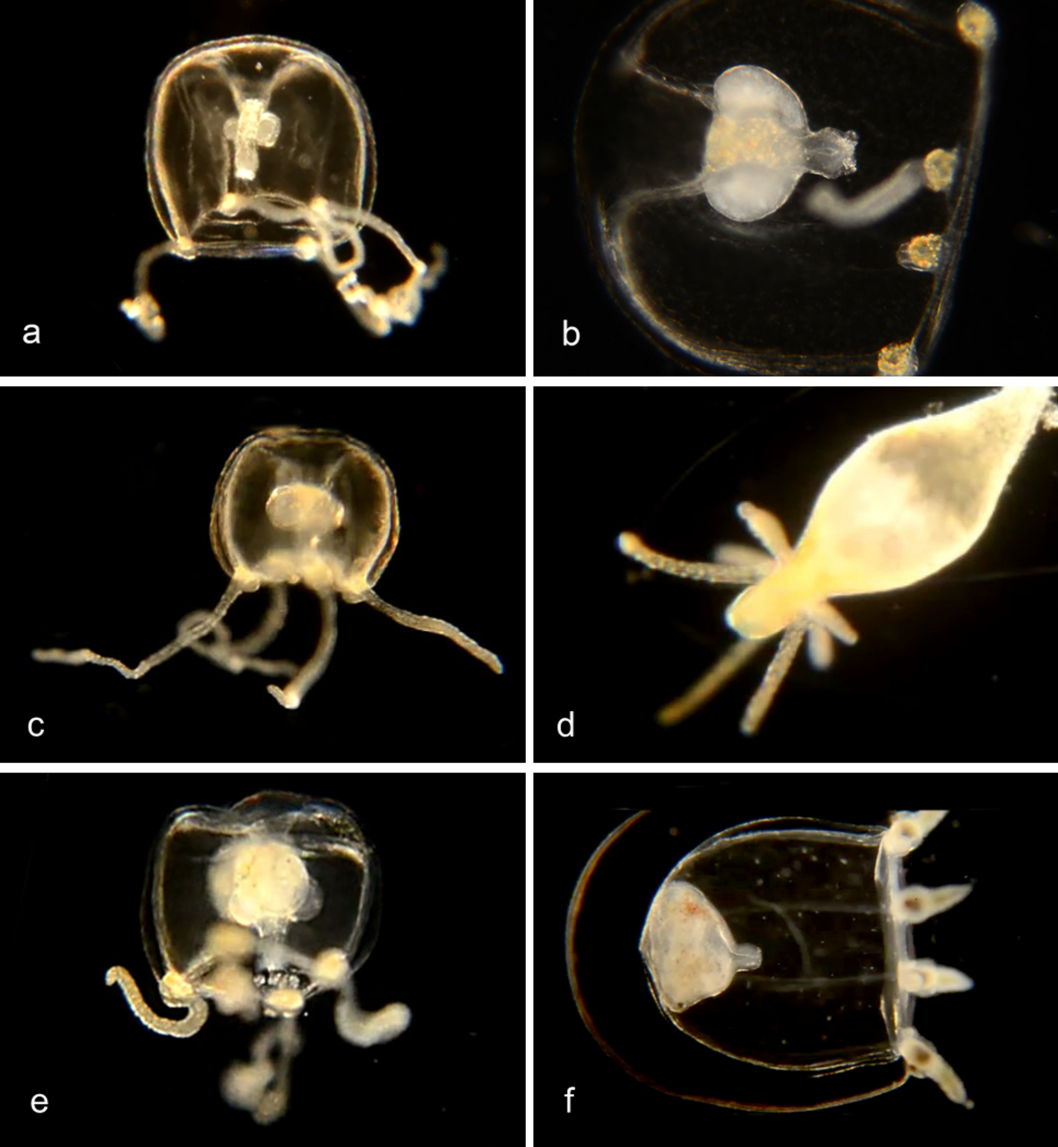
Species with four single tentacles. a,b. *Bougainvillia sp*. 1PJB (Bin ACR4341): Medusa with four radial canals and four single tentacles; very short unbranched oral tentacles present. Material collected: 16 medusae (11 off Newport Pier, 2 off Balboa at Coral, 3 from Ocean off Dana Point). a, Medusa BIOUG01227-E08. b, Medusa BIOUG01213-C03. c,d,e. *Bougainvillia sp*. 2PJB (BIN ACR4343): Medusa with four radial canals and four single tentacles, and four very short unbranched oral tentacles. Material collected: 27 medusae (10 off Newport Pier, 12 off Balboa at Coral, one in Delhi Channel, 2 in Ocean off Crystal Cove, one in Ocean off Newport, one in Harbor entrance), one hydroid off Newport Pier. c, Medusa BIOUG01227-D05. d, Hydroid BIOUG01227-A07. e, Manubrium with medusa buds and short unbranched oral tentacles BIOUG19284 H06; f, *Bougainvillia sp*. 3PJB (BIN ACW4756): Four radial canals, four unbranched tentacles, no oral tentacles. Material collected: One medusa BIOUG01227-G02, off Newport Pier.

**Fig 3:**
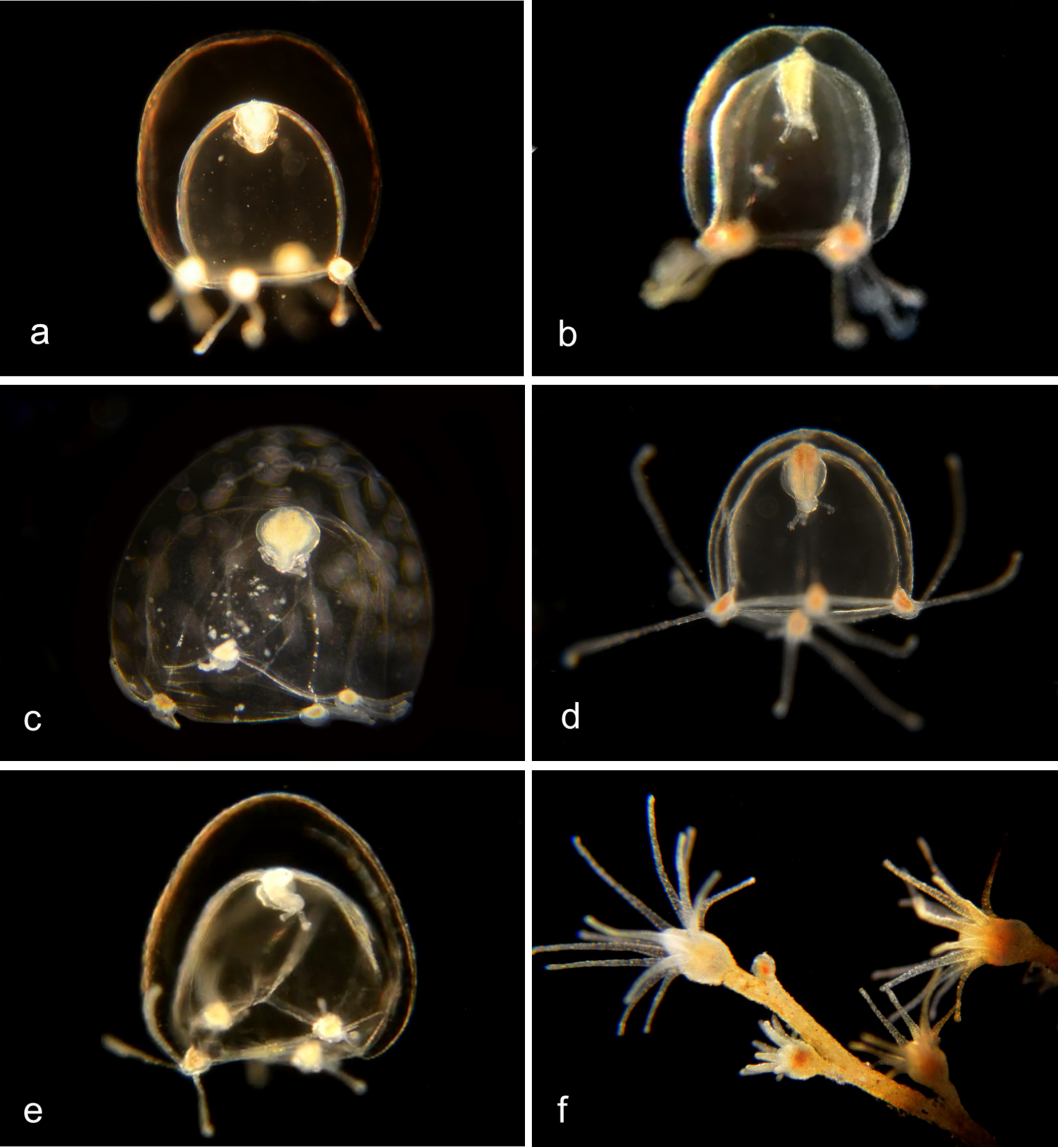
Species with four pairs or triplets of tentacles. a. *Bougainvillia muscus* (BIN ACO8375). Medusa with four radial canals, four pairs of tentacles and four short unbranched oral tentacles. Material collected: Two medusae, off Lido Island. These show a close match (>98%) by DNA barcode with specimens of *Bougainvillia muscus* collected in Skagerrak (Sweden), Roscoff (France) and New Brunswick (Canada). BIOUG01213-H09. b. *Bougainvillia sp*. 4PJB (BIN ACR4338). Medusa with four radial canals, four pairs of tentacles and four short unbranched oral tentacles. Material collected: 7 medusae, all off Newport Pier. BIOUG01213-G06. c. *Bougainvillia sp*. 5PJB (BIN ADC0389). Medusa with four radial canals, four pairs of tentacles and four short oral tentacles. Material collected: One medusa off Balboa at Coral. BIOUG19285-A03. d. *Bougainvillia sp*. 6PJB (BIN ACR 4342). Medusa with four radial canals, four triplets of tentacles and four oral tentacles. Material collected: Three medusae (1 off Newport Pier, two off Lido Island) BIOUG01227-C08. e,f. *Bougainvillia sp*. 7PJB (BIN ACR 4339): Medusa with four radial canals, four triplets of tentacles, and two unbranched oral tentacles. Material collected: One medusa (off Crew Dock), one hydroid colony (off NAC). a, Medusa BIOUG01213-D09; b, Hydroid BIOUG19284-F02.

Genus ***Bougainvillia*.** Medusa with four radial canals and four single or bundled marginal tentacles; gonads on the side of the manubrium [8,9] (Figs 2 and 3). All *Bougainvillia* species described by Vannucci and Rees [8] except *B*. *multicilium* and *B*. *prolifera* have dichotomously branching oral tentacles [8], which is not the case for the species described here. *B*. *multicilium* and *B*. *prolifera* have 10–12 or five tentacles respectively on each marginal bulb [8], which is also not the case for the species described here. We therefore provide tentative species names (nPJB) for most of the species described here.

**Fig 4:**
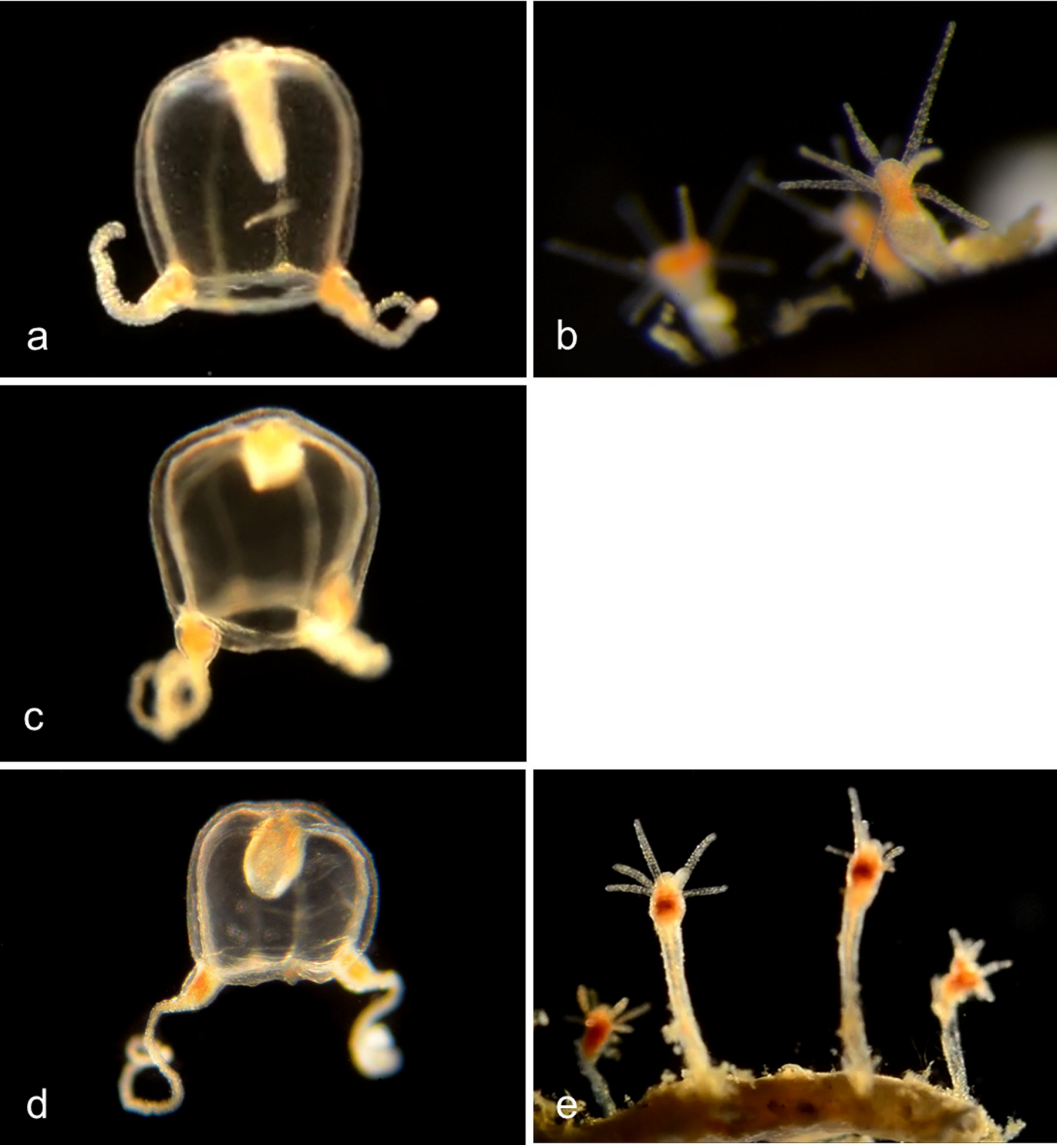
*Amphinema* species. a,b. *Amphinema sp*. 1PJB (BIN ACR4255): Material collected: four medusae (3 off Newport Pier, one from Harbor entrance), three hydroid colonies, all from off Balboa Pavilion dock. a, Medusa BIOUG19284-G12; b, Hydroid BIOUG19285-D12. c. *Amphinema dinema* (BIN ACW2490): Material collected: One medusa, off Newport Pier. 100% match of DNA barcode COI with a specimen from Roscoff, France [9]. Medusa BIOUG01227-H02. d,e. *Amphinema sp*. 2PJB (BIN ACR4184): Material collected: Four medusae (Two off Balboa Pavilion Dock, one from Bayside Marina, one off Newport Pier), three hydroid colonies (two off Newport Pier, one off Balboa Pavilion Dock) d, Medusa BIOUG01227-H10; e, Hydroid BIOUG19285-D07.

*Genus* **Amphinema.** Medusa with four radial canals and two single tentacles, no oral tentacles; Hydroids with a conical proboscis and a single whorl of simple tentacles [9] (Fig 4).

#### Family Cladonematidae

**Fig 5:**
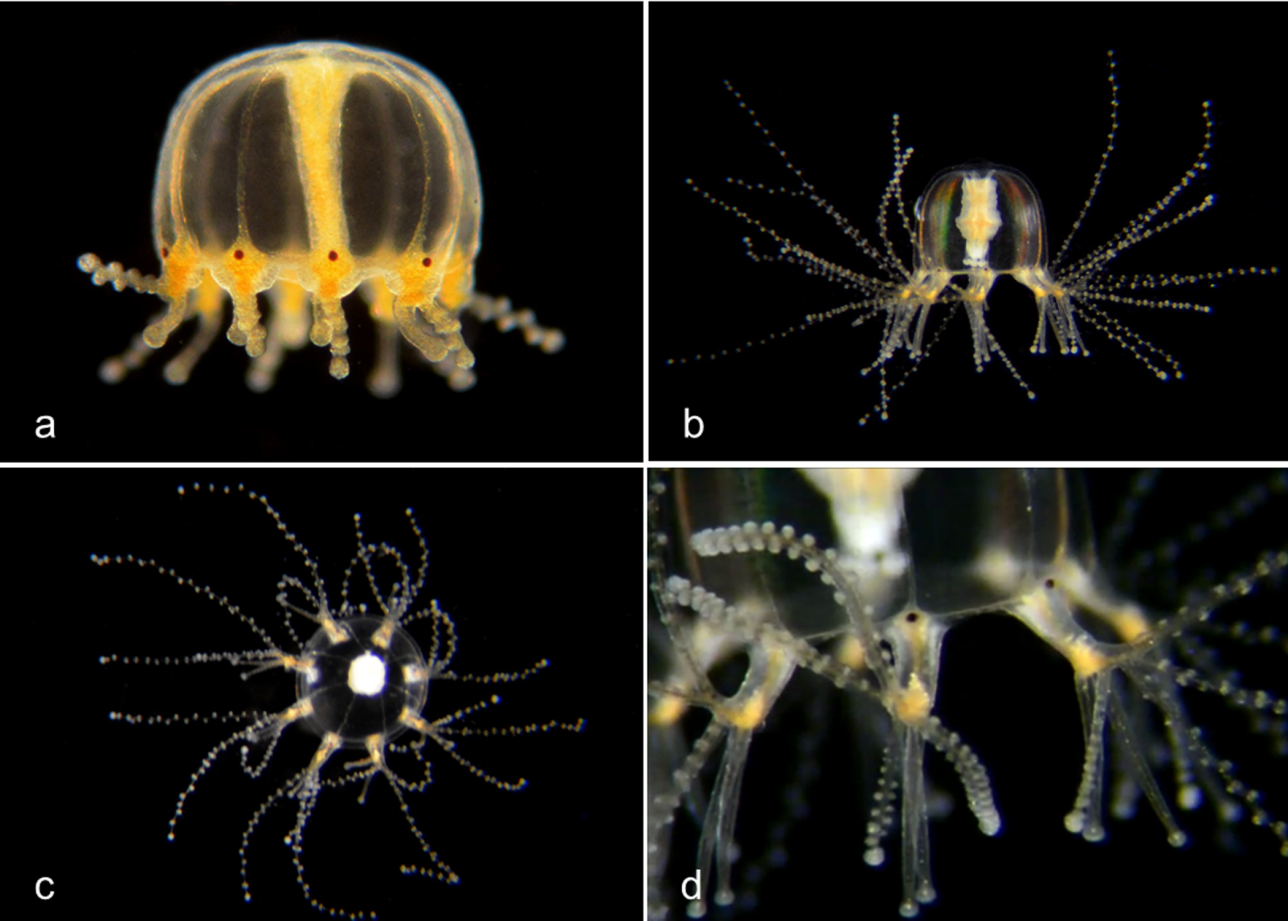
Cladonema californica. a, Medusa BIOUG19284-C05. b, c, d, This specimen was found in tanks at Orange Coast College, Orange County, CA on 7/19/2018. Hydroid stage not yet found locally.

The family Cladonematidae, represented by the genus ***Cladonema***, is sometimes collected in plankton samples, but its habitat is reported to be on the leaves of eelgrass, where the inner branches of all nine tentacles are used as suckers and the outer branches carry stinging cells. At the base of each tentacle is a red ocellus (eye-like structure) [10,11]. Material collected (Fig 5; BIN ACR4340): 7 medusae (3 off Lido Island; two off Balboa at Coral, one off Back Bay Science Center Dock, one off Crew Dock).

#### Family Corymorphidae

**Fig 6:**
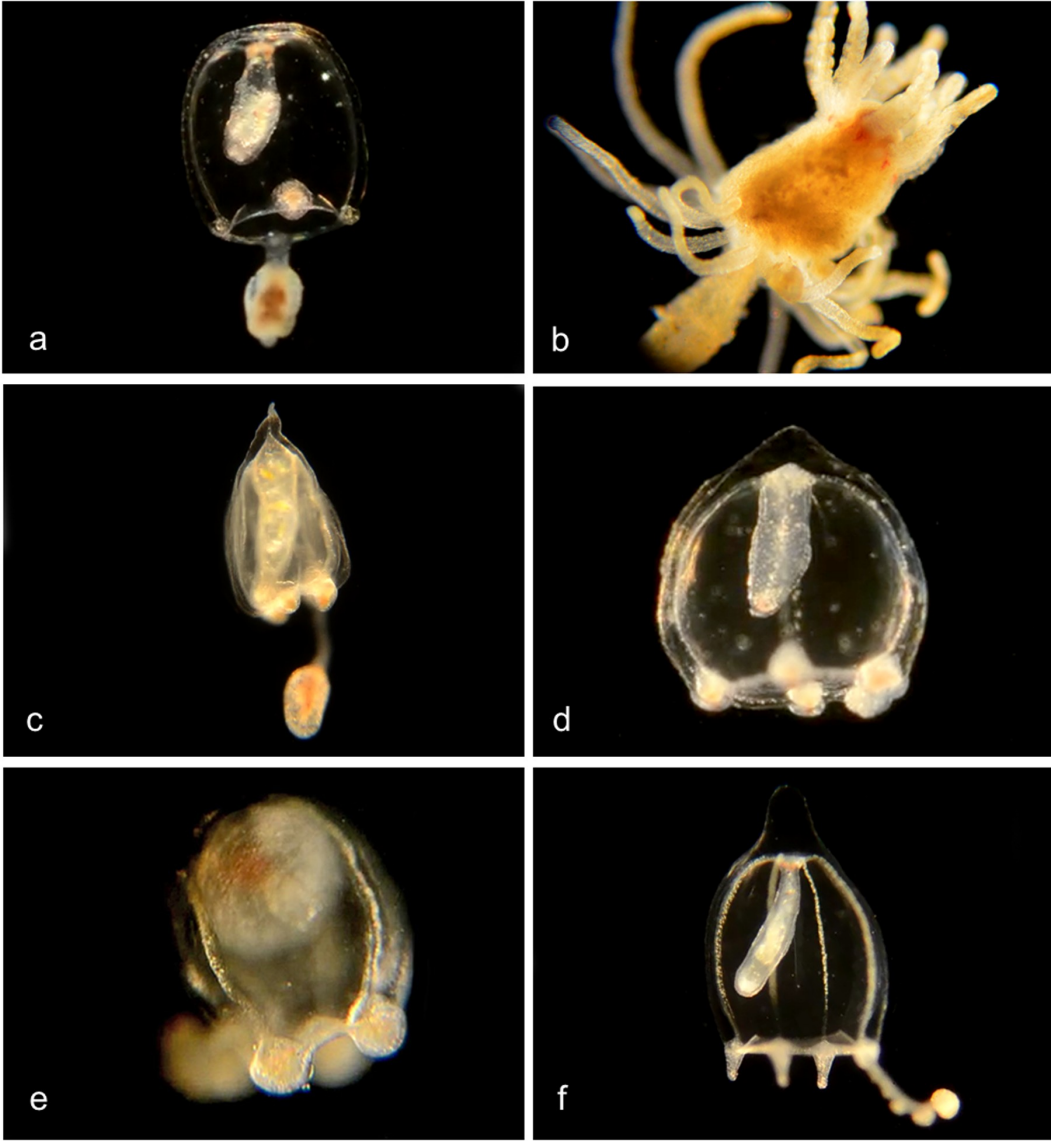
*Corymorpha* species. a, b. *Corymorpha sp*. *1PJB* (BIN ACR4296). Material collected: 13 medusae (3 at Crew Dock, 3 at Balboa at Coral, 3 at Lido, and one each at NAC, Delhi channel, Newport Pier and Bayside Marina); two hydroids (one at Balboa at Coral, one at Crew Dock). a, Medusa BIOUG19284-H03; b, Hydroid BIOUG19289-A08. c. *Corymorpha sp*. *2PJB* (BIN ADO3522). Material collected: 1 medusa, at Harbor entrance. Medusa CCDB24005-H02. d. *Corymorpha sp*. *3PJB* (BIN ACZ1499). Material collected: 3 medusae (1 at Lido at Genoa, 1 at Crew Dock, 1 at Bayside Marina). Medusa BIOUG19284-E04. e. *Corymorpha sp*. *4PJB* (BIN ACH5005) close DNA match to both *C*. *bigelowi* and *C*. *verrucosa*. Material collected: 1 medusa (at Lido at Genoa). Medusa BIOUG01213-G01. f. *Corymorpha bigelowi* (BIN ACH5002). [13]. Material collected: 21 medusae (8 off NAC, 4 from Delhi Channel, 4 off Crew Dock, 2 off Newport Pier, 2 off Lido Island at Genoa, and 1 off Balboa Island at Coral), no hydroids. Medusa BIOUG19284-H07.

In the genus ***Corymorpha*** [12] the medusa is distinguished by having one of the four tentacles much longer than the others and containing most or all of the stinging cells. Four radial canals, no oral tentacles. The hydroid has one ring of tentacles around the mouth and another ring more basally (Fig 6).

#### Family Corynidae

**Fig 7:**
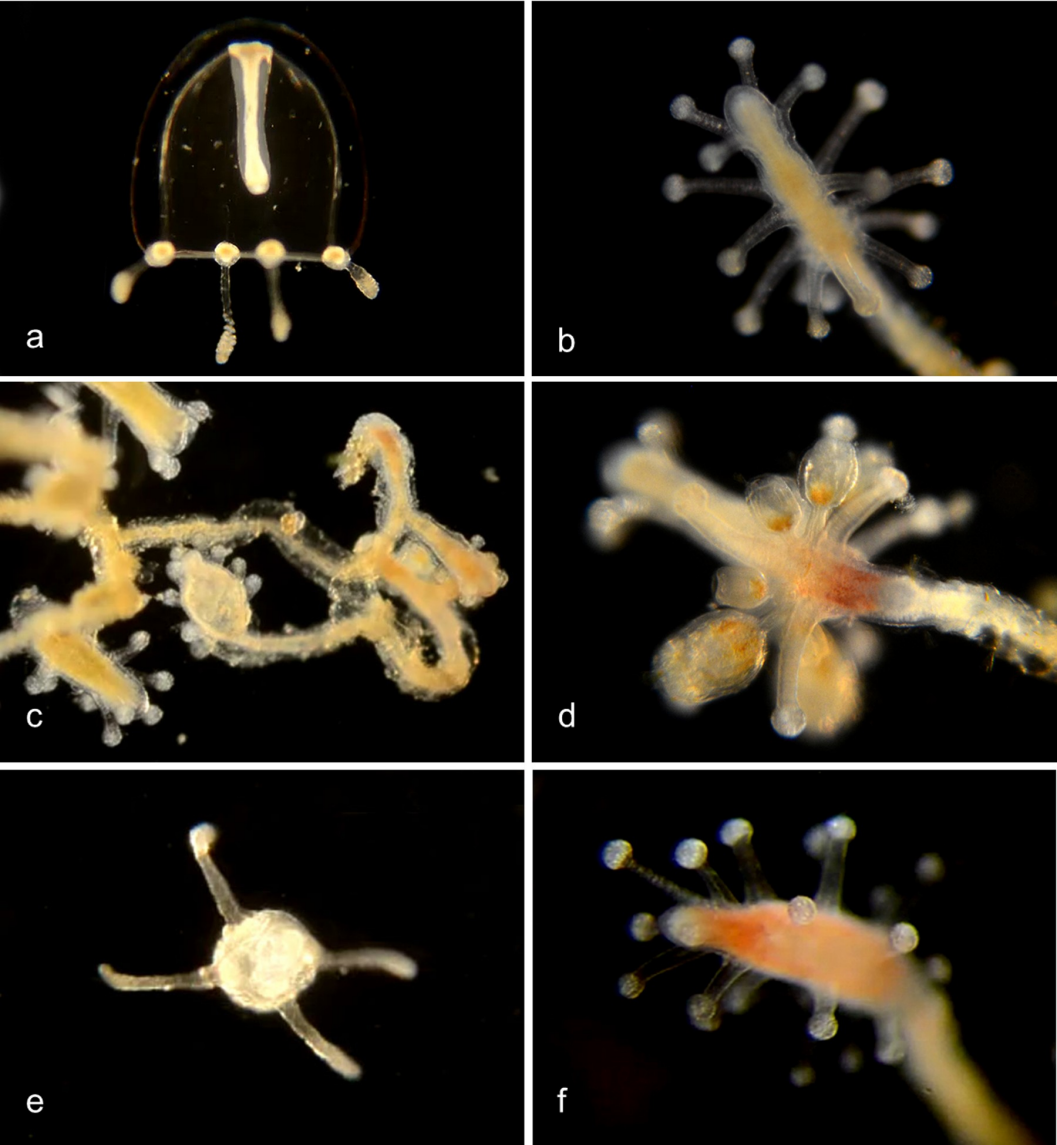
*Coryne* species. **a-e.***Coryne eximia* (BIN ACR4295). Material collected: 17 medusae (9 at Newport Pier, 5 at Balboa at Coral, 1 each at Crew Dock, Balboa Pavilion dock, and Reef Point CCSP), 8 hydroids (4 at Newport Pier, 2 at CCSP and 1 each at Mussel Rocks and Reef Point, CCSP) and one actinula larva off Newport Pier. These 26 specimens show closely matched DNA barcodes with one another but with no other sequences in BOLD or Genbank. One other (CCDB 24005 C11 off Balboa at Coral) shows a closer match with one specimen from Norway and one from South Africa, and one more (BIOUG01213 F05 off Newport Pier) shows a closer match with a specimen from Chile. a, Medusa BIOUG01227-A09. b, Club Hydroid BIOUG19289-E06. c, Club hydroid BIOUG19289 F01. d, Club hydroid BIOUG19289-E05. The medusa buds arise in the tentacle axils. e, Actinula larva BIOUG01227-H04. f, *Coryne uchidai* (BIN ACR4087) Club hydroid BIOUG01213-G02: Material collected: 1 hydroid at Lido at Genoa. DNA barcode shows 98.77% match with a specimen from Hokkaido, Oshoro, Japan.

Genus ***Coryne*.** Medusa has four radial canals and four tentacles with nematocysts concentrated at the tips; long manubrium with no oral tentacles. Stolon is branched, hydroids club-shaped with scattered knob-shaped tentacles carrying clusters of nematocysts at their tips (Fig 7) [14].

#### Family Oceaniidae

**Fig 8:**
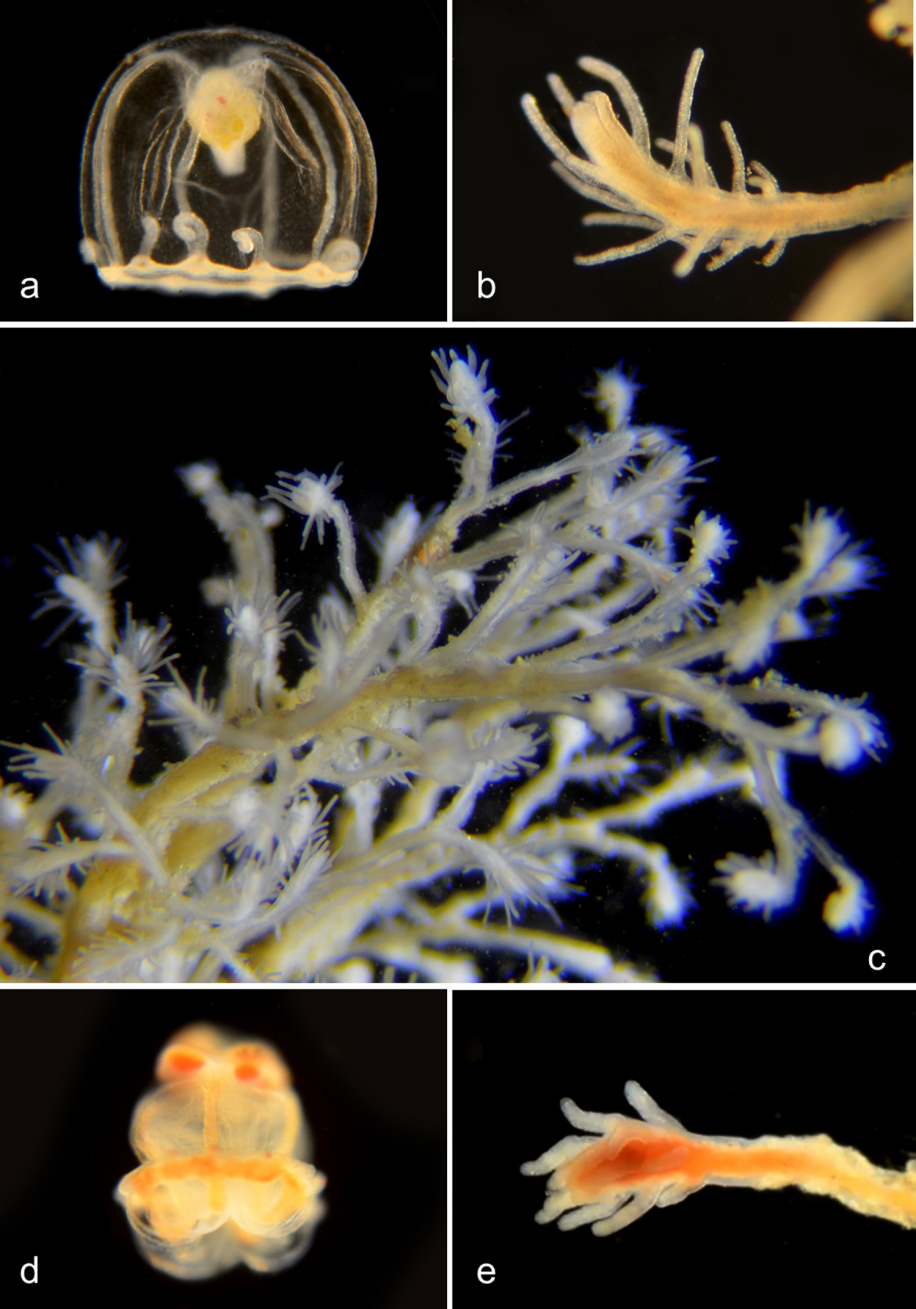
***a-c*. *Turritopsis dohrnii* (BIN ACR4293)**. Material collected: 8 medusae (2 off NAC, 2 off Balboa at Coral, 3 off Lido at Genoa, one off Balboa Pavilion); one hydroid (off Pavilion Dock). a, Medusa BIOUG01227-C01; b, Club hydroid BIOUG19285-E09; c, Club hydroid BIOUG19285-E09. **d,e. *Turritopsis nutricula* (BIN ACI0369).** Material collected: d, One medusa (inverted?) BIOUG19285-G08 (from Newport Harbor entrance); e, Club hydroid CCDB-25431-F11 (Off Newport Pier).

Genus *Turritopsis* (Fig 8) [15–17]. Medusa with 4 radial canals and 8–12 tentacles (80–90 in literature); manubrium with no oral tentacles. Hydroid with filiform tentacles scattered over much of the body.

#### Family Porpitidae

**Fig 9:**
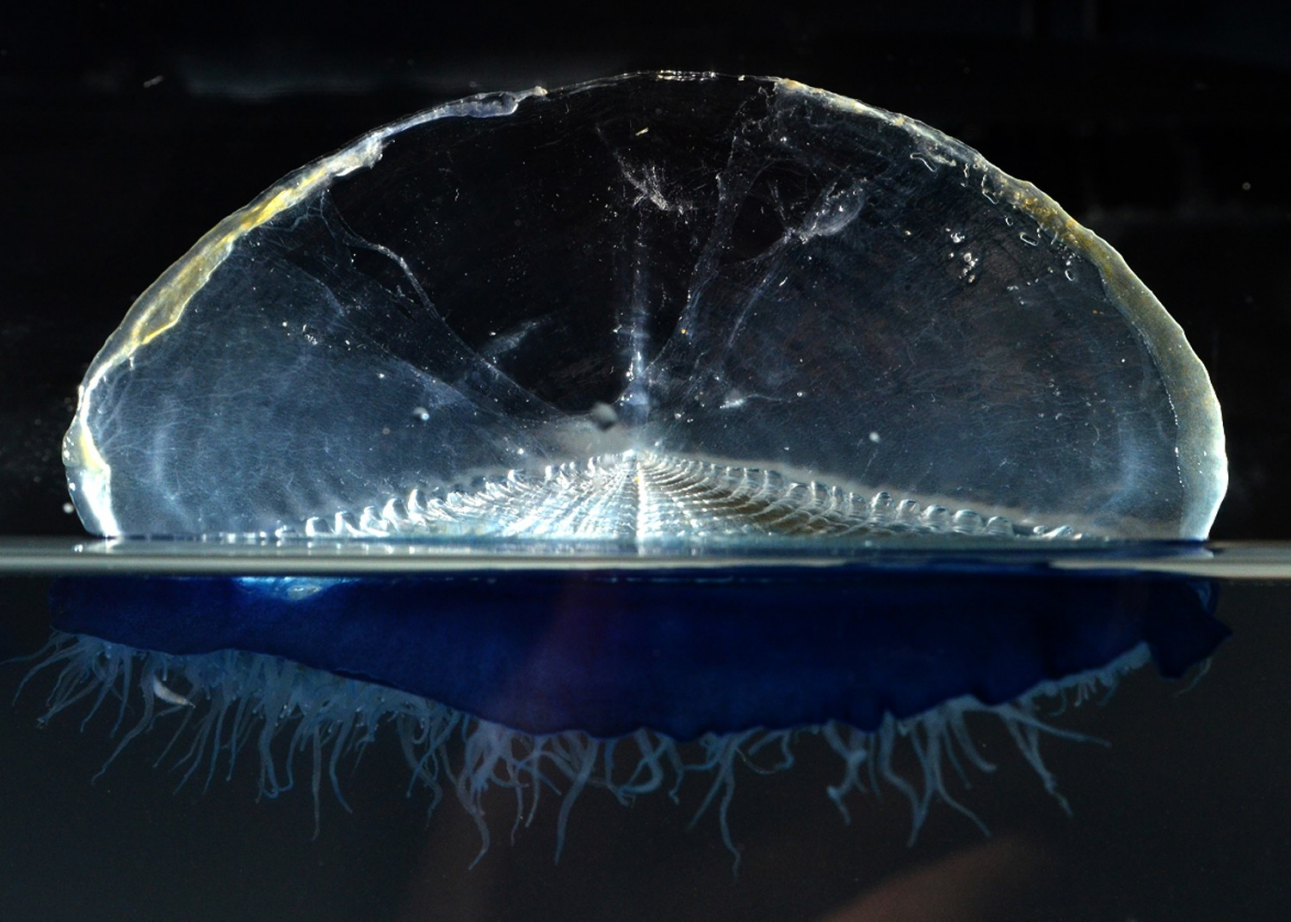
By-the wind Sailor, *Velella velella*. Material collected: One colony, from San Clemente Beach.

By-the wind Sailor, ***Velella velella*** (BIN ACR4084) (Fig 9). [18]. Each of these floating jellies is a colony, composed of many individuals that are specialized for various functions: the gonozooids carry out feeding and reproduction, and the dactylozooids protect the colony using stinging cells. The stiff sail catches the wind and propels the colony at a slight angle from directly downwind over the ocean surface. Under some conditions, thousands of them wash ashore on beaches.

#### Family Tubulariidae

**Fig 10:**
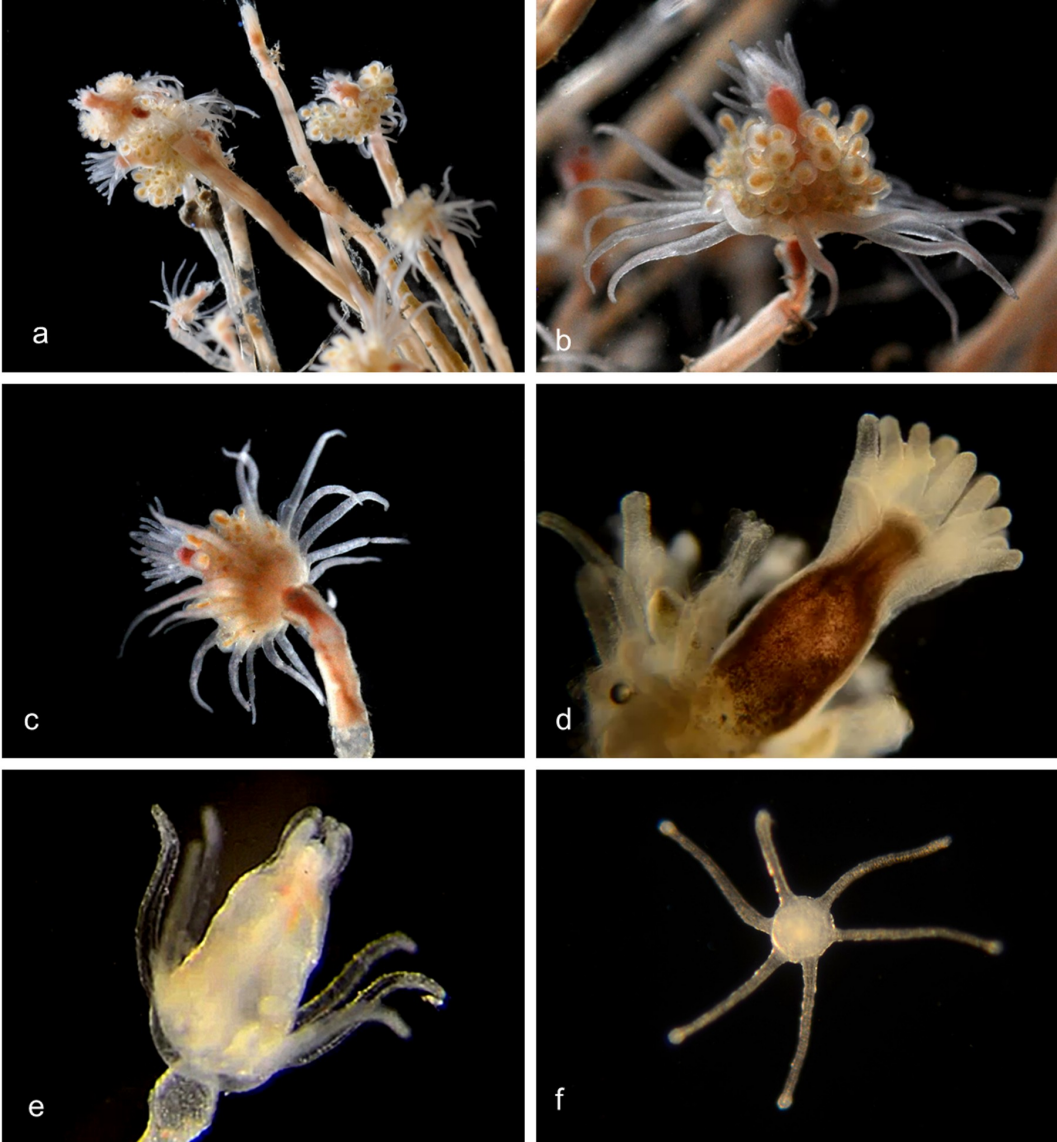
Pink-hearted hydroid, *Ectopleura crocea*. a, Hydroid colony (not barcoded); b, Hydroid (not barcoded); c, Hydroid (not barcoded); d, Hydroid BIOUG01227-C07; e, Hydroid BIOUG01213-G03; f, Actinula larva BIOUG01213-E05.

Pink-hearted hydroid, ***Ectopleura crocea*** (BIN ACH9225) (Fig 10). [12, 19]. Exotic species, native to the east coast of North America. It lacks a medusa stage, but grows as colonies of hydroids, each of which has two whorls of tentacles. Medusa buds (“medusoids”) develop between the two whorls of tentacles, but are not released. They produce either eggs or sperm, and internal fertilization occurs in the female medusoids. The fertilized eggs develop into actinula larvae, which are released and develop directly into hydroids. Material collected: 3 actinula larvae (2 off Newport Pier, one off NAC), 5 hydroids (2 off Newport Pier, one off Balboa at Coral, one from Ocean, one from Harbor entrance).

### Order Leptothecata: With Thecate hydroids

These have both medusa and hydroid stages, and the medusae are usually larger than in the Anthoathecata. The hydroid stalk is thecate (surrounded by an acellular sheath). The medusae of various species are difficult or impossible to distinguish from one another, so the taxonomy is based on the hydroid phase (the opposite to the usual situation in Anthoathecata).

#### Family Campanulariidae

Notable genera are *Clytia*, *Obelia* and *Orthopyxis* [20]. The hydroid colony reproduces asexually. During the hydroid stage of the life cycle, colonies are attached to substrate) surfaces. A mature colony bears feeding hydroids (gastrozooids), defensive hydroids, and reproductive hydroids (gonozooids), the last producing medusae by budding. The umbrella-shaped medusa has four or more unbranched tentacles, and the gonads are on the radial canals.

**Fig 11:**
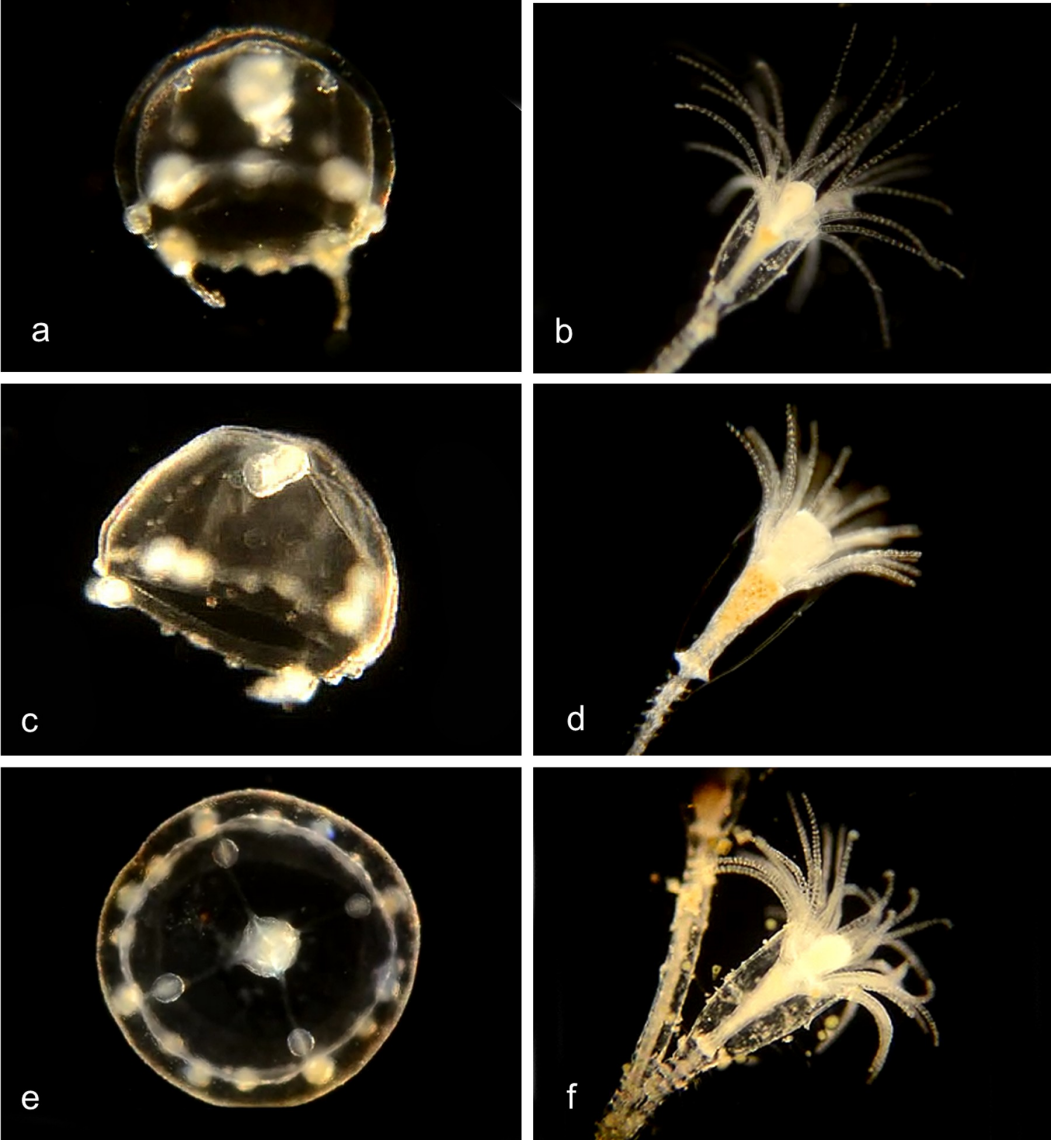
Clytia species. a. *Clytia elsaeoswaldae* (BIN ACR4294). Material collected: 2 medusae (off Balboa at Coral) as well as three medusae from Bahia de los Angeles, Mexico. BIOUG19284-D07. b. *Clytia hemisphaerica* (BIN AAD5403). Material collected: One hydroid BIOUG01227-E11 off Balboa at Coral. c,d. *Clytia gracilis* (BIN AAD5401). Material collected: One medusa off Newport Pier, one hydroid off Reef Point, Crystal Cove State Park. c, Medusa BIOUG01213-D07; d, Hydroid BIOUG19289-C05. e,f. *Clytia sp*. *701 AC* (BIN AAR9451). Medusa phase: Four tentacles, four developing tentacles, and eight statocysts. Material collected: 3 medusae off Dana Point, one off Lido at Genoa; One hydroid off Balboa at Coral). e, Medusa BIOUG19284-C12; f, Hydroid BIOUG01213-G11.

Genus ***Clytia***[21,22] (Fig 11)

#### Genus *Obelia*

**Fig 12:**
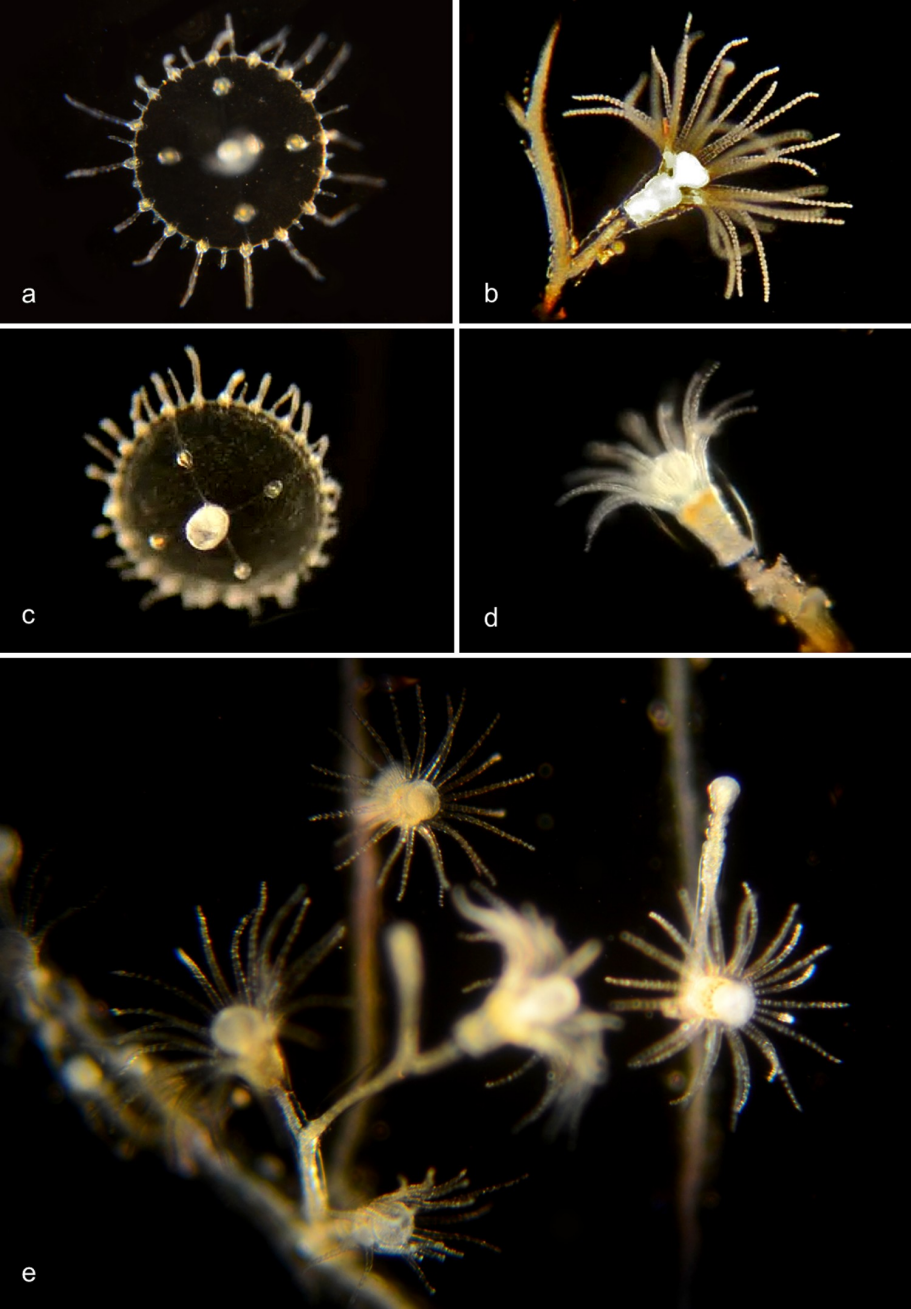
*Obelia* species. a,b. *Obelia dichotoma* (BIN ACO3910). Material collected: 5 medusae (3 off Newport Pier, one off Balboa at Coral, one off BBSC); 6 hydroids (5 off Newport Pier, one off Reef Point, CCSP). a, Medusa CCDB-25433-B12; b, Hydroid CCDB-24005-A10. *Obelia sp*. 1PJB (BIN ACR4187). Material collected: 15 medusae (2 offshore from CCSP, 2 at NAC, 7 offshore from Dana Point, 2 from Harbor entrance, 1 off Balboa at Coral and one off PCH bridge); 10 hydroids (4 off Newport Pier, 5 off Balboa at Coral, one from Harbor). c, Medusa BIOUG012213-E12; d, Hydroid BIOUG01213-G10. *Obelia geniculata* (BIN AAA708). Material collected: One hydroid, off Newport Pier. e, Hydroid BIOUG19284-G10.

According to Cornelius [23] the medusae of this genus are indistinguishable from each other, and over seventy species had been described from the hydroid stage between 1830 and 1948. Cornelius referred all of these specimens to only three nominal species: *O*. *bidentata*, *O*. *dichotoma*, and *O*. *geniculata*. The identification characters given by Cornelius cannot all be used on our records, since they refer to growth habit and substrate, which has not always been recorded, or to the morphology of the hydrothecal rim which is usually not visible in our unstained specimens. Our DNA barcoding data identify three species (Fig 12), of which one is clearly *O*. *dichotoma* and one is *O*. *geniculata*. The third group (*Obelia* sp. 1PJB) is presumably *O*. *bidentata*, although we have not been able to confirm that from the identification characters, since the rim of the theca is not visible in our unstained live specimens.

#### Genus *Orthopyxis*

**Fig 13:**
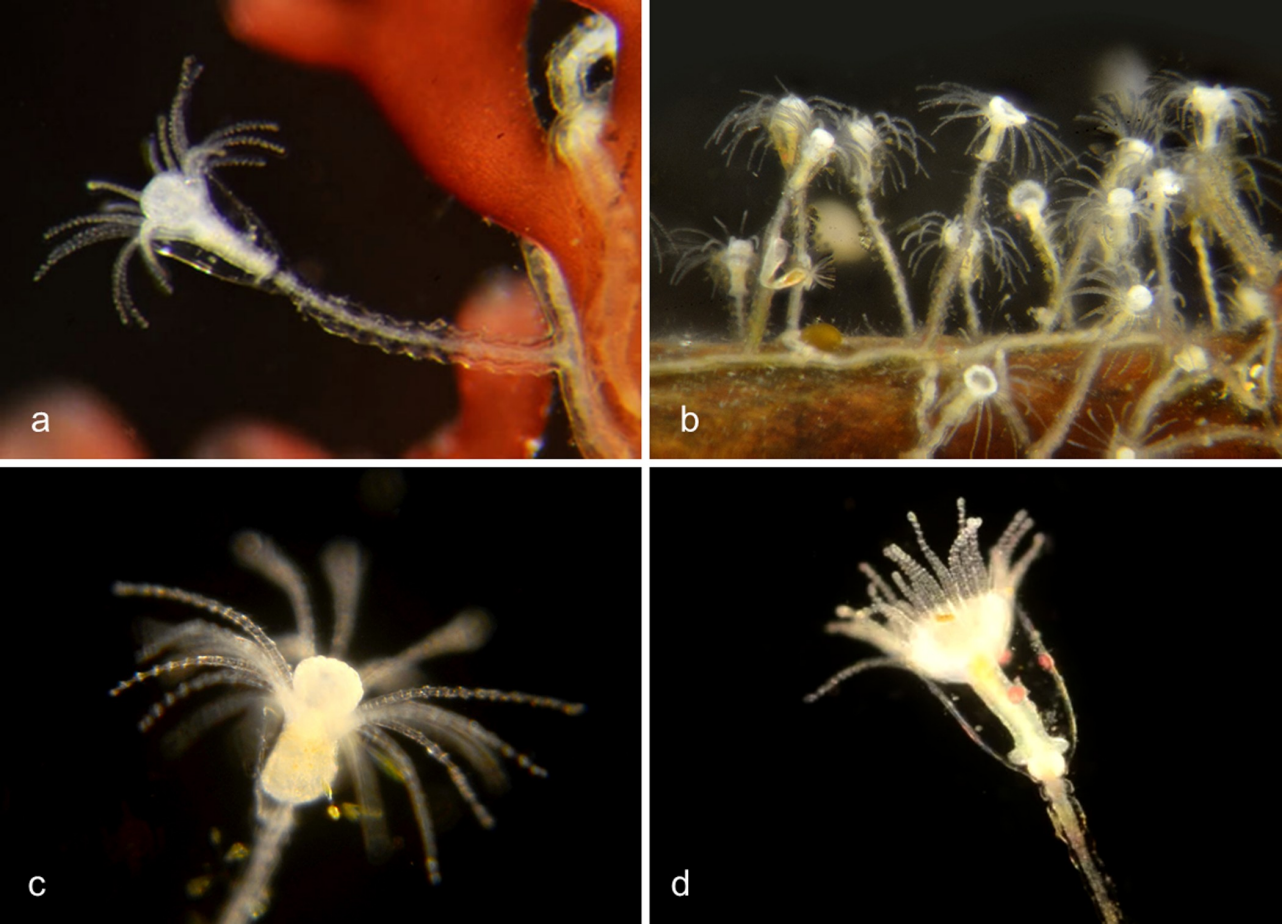
*Orthopyxis* species. a. *Orthopyxis everta* (BIN ADE2083). Material collected: One hydroid off Reef Point, Crystal cove State Park. Hydroid BIOUG19289-E01. b. *Orthopyxis sp*. 1PJB (BIN ADE2082). Material collected: One hydroid off Mussel Rocks, Crystal Cove State Park Hydroid BIOUG19289-D09. c. *Orthopyxis sp*. 2PJB (BIN ACZ1022). Material collected: One hydroid off Newport Pier. Hydroid BIOUG19284-E01. d. *Orthopyxis sp*. 3PJB (BIN ADH3873). Material collected: One hydroid from ocean off Newport Beach. Hydroid CCDB-25431-G09.

The stalk of the hydroid is annulated and has a smooth rim (Fig 13). This genus [24] produces eumedusoids (incomplete medusae, generally lacking tentacles, that may or may not be released) rather than medusae [20], but we have not found these.

#### Family Lovenellidae

**Fig 14:**
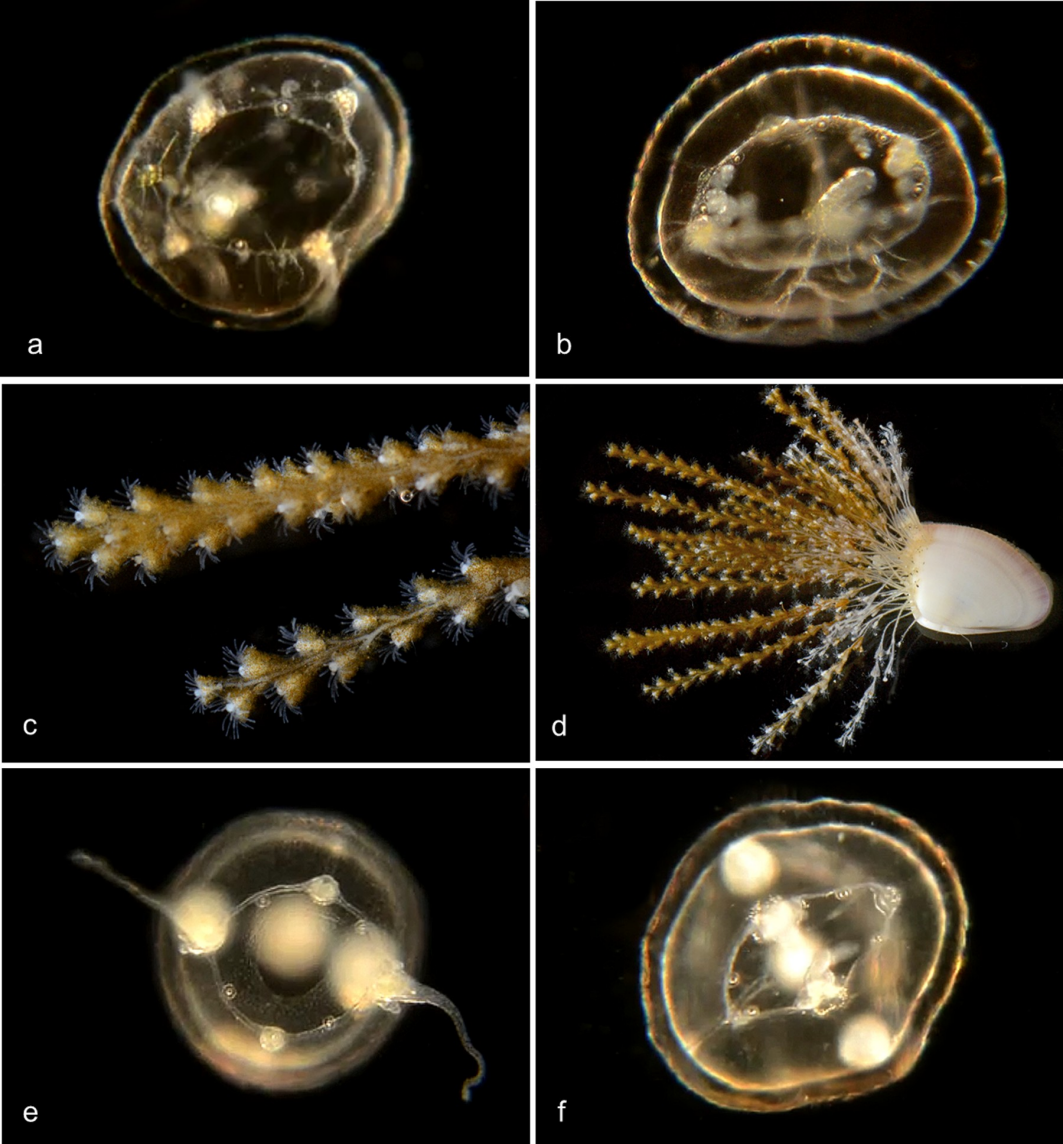
Genus *Eucheilota*. a. Close to *Eucheilota bakeri* (BIN ACW 1912) [25]. 4 statocysts. Material collected: 5 medusae (2 at Newport Pier, 1 at Newport Aquatic Center, 2 at Balboa at Coral). Medusa BIOUG19284-D02 (Ventral). b. *Eucheilota sp*. (BIN ACR4297). 8 statocysts. Material collected: 3 medusae (1 at Crystal Cove, 2 at Newport Pier). Medusa BIOUG01227-E06. c-f. *Eucheilota bakeri* (BIN ACR4560) [25]. Material collected: Two hydroid colonies, attached to Bean Clam, *Donax gouldii*, washed up on the beach under Newport Pier. c, Hydroid BIOUG0213 C11; d, Hydroid BIOUG0213 C11; two medusae (each with 4 statocysts), one from the Harbor entrance, one off Balboa at Coral. e, Medusa CCDB 24005 C03: f, Medusa CCDB-25431-H05.

Medusae with short manubrium; without gastric peduncle; 4 straight radial canals; gonads on radial canals separated from stomach; marginal tentacles hollow; 4, 8 or 12 statocysts. Genus ***Eucheilota*** (Fig 14).

Subclass Trachylina

### Order Actinulida

No material collected.

### Order Limnomedusae

No material collected.

### Order Narcomedusae

Usually reproduce sexually as medusae, and do not form hydroids. The medusa) has a dome-shaped bell with thin sides. The tentacles are attached above the margin of the bell with usually a gastric pouch above each. There are no bulbs on the tentacles and no radial canals.

#### Family Solmarisidae

**Fig 15:**
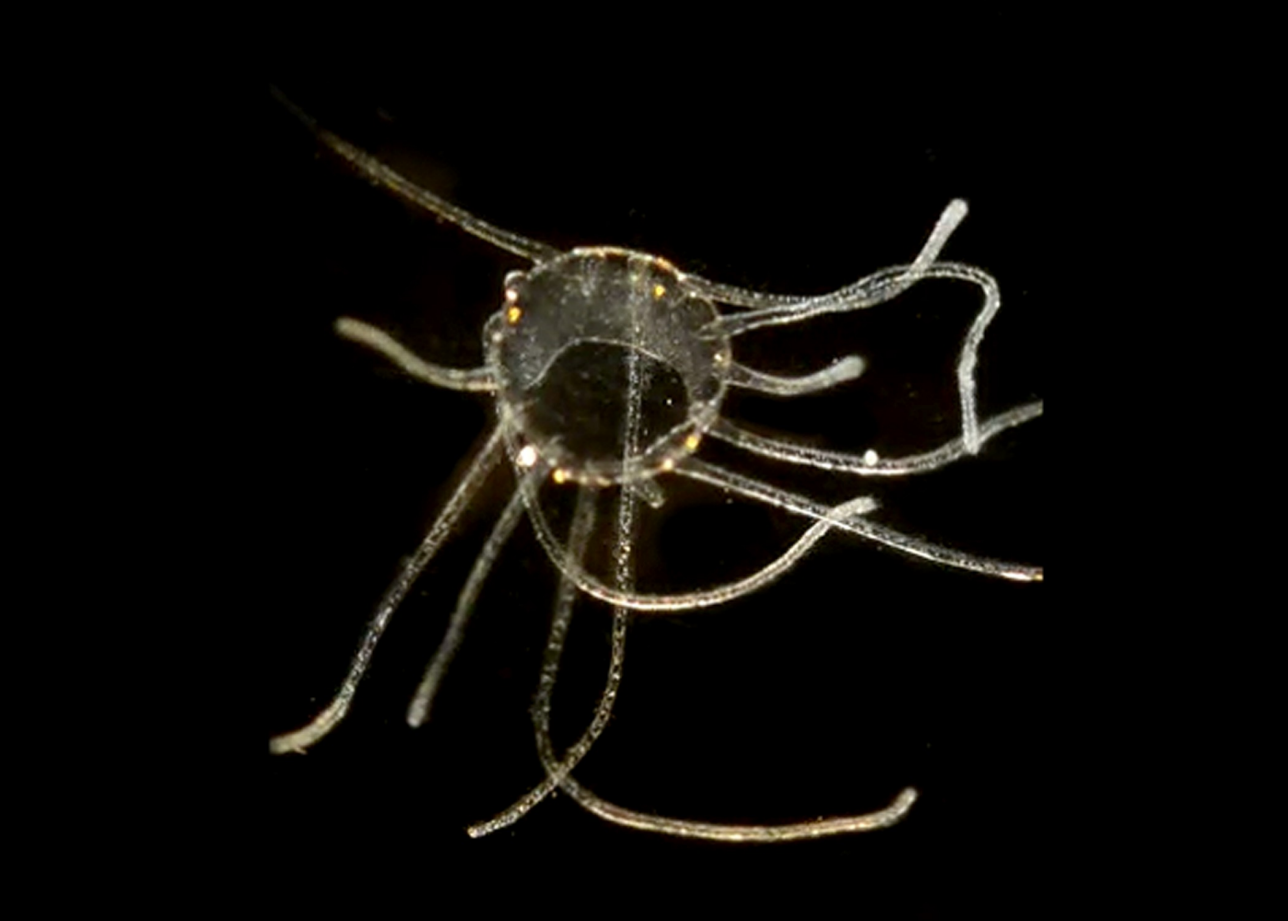
Medusa CCDB-25431-D07. Collected from Newport Harbor.

One medusa (Fig 15) representing this family [26] was collected from Newport Harbor, but it was not DNA barcoded.

### Order Trachymedusae

Reproduce sexually as medusae and do not form hydroids.

#### Family Geryoniidae

**Fig 16:**
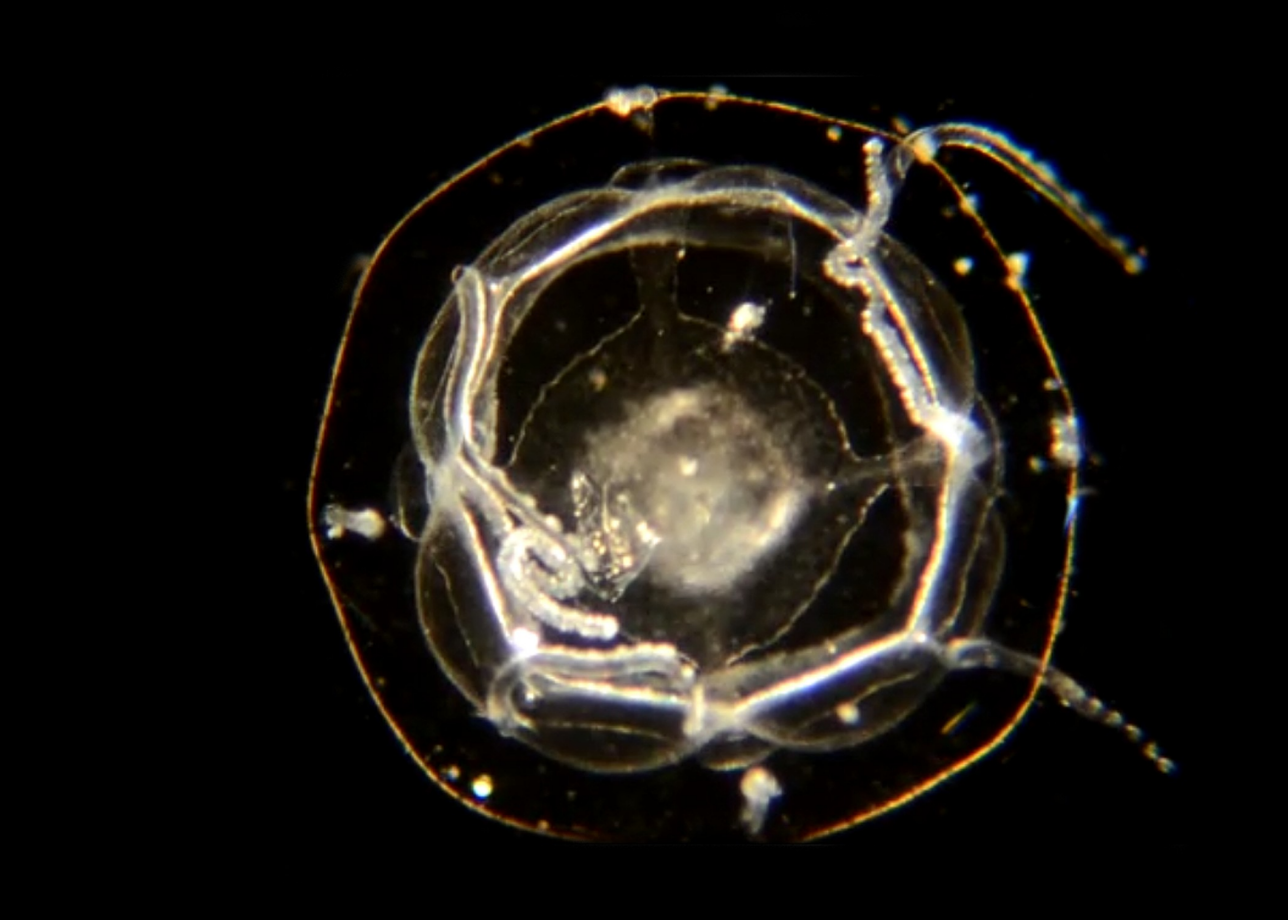
*Liriope tetraphylla*: BIN ACH9275. Material collected: 9 medusae (4 off Newport Pier, 2 off Dana Point, and one each from Balboa at Coral, Newport Harbor, and off CCSP). Medusa BIOUG01227-A02.

***Liriope tetraphylla*.** Medusa (Fig 16) has a nearly hemispherical umbrella with four hollow perradial marginal tentacles, and four solid interradial tentacles [27]. The ring canal is broad, there are four straight radial canals and the manubrium extends from a long gastric peduncle. This species does not have a hydroid phase.

## Discussion

In the order Anthoathecata (athecate hydroids), DNA barcoding allowed for the discrimination between the medusae of seven putative species of *Bougainvillia*. Of these, three species had medusae with four single tentacles, three (including the previously described *B*. *muscus*) with four paired tentacles, and two with four triplets of tentacles. The hydroid stages were documented for two of the *Bougainvillia* species.

We documented the medusa (with two simple tentacles) of three putative species of *Amphinema*, (including the previously described *A*. *dinema*) and documented the hydroid stages for two of them. DNA barcodes were obtained for medusae of one species of *Cladonema*, five putative species of *Corymorpha* (including the previously described *C*. *bigelowi)* with the matching hydroid phase for one; and *Coryne eximia*, *Turritopsis dohrnii* and *Turritopsis nutricula* McCrady with the matching hydroid phases for all of them. We identified the hydroid phase of *Coryne uchidai* by its DNA barcode. The hydroid and actinula larvae for the pink-hearted hydroid *Ectopleura crocea* were identified and matched by DNA barcoding.

In the order Leptothecata (thecate hydroids) medusae were identified for *Clytia elsaeoswaldae*, *Clytia gracilis* and *Clytia sp*. *701 AC* and paired with the hydroid phases for the latter two species. Medusae were matched with the hydroid phases for two species of *Obelia* (including *O*. *dichotoma*) and *Eucheilota bakeri*. *O*. *geniculata* was collected as a single hydroid. Four species of *Orthopyxis*, (including *O*. *everta*), were collected only as hydroids.

One member of the family Solmarisidae, representing the order Narcomedusae, and one member (*Liriope tetraphylla*) of the order Trachymedusae were documented as medusae.

The DNA differences in the COI barcode are, of course, probably not responsible for the morphological differences we have observed between specimens in separate taxa. However, the DNA differences between morphologically distinct medusae confirm that the specimens represent different taxa rather than different developmental stages within species. In some cases (e.g. *Bougainvillia*, *Amphinema*, *Liriope tetraphylla*, *Obelia*) DNA barcoding suggests that currently recognized species may include unrecognized distinct taxa, which are not obviously different at the morphological level.

A similar example shows molecular species boundaries in the absence of morphological, ecological or geographical differentiation in the Red Sea octocoral genus *Ovabunda* [28].

We also included some members of the class Anthozoa in the early stages of this study, and confirmed other reports that the use of DNA barcoding with this group is more problematic than with hydrozoans. Among local species, *Anthopleura elegantissima* and *A*. *sola* show identical DNA barcodes and *A*. *xanthogrammica* is very similar (unpublished information). We assume that this reflects very recent separation of these species.

The use of the DNA Barcode allows matching of hitherto unrecognized life-cycle stages within individual species, avoiding the ambiguities that have previously led to the medusa and hydroid stages being assigned to separate species. Hydroid and medusa phases were originally matched by “circumstantial evidence” [29] and later by culturing hydroid phases in the laboratory and observing the release of medusae [30,31]. DNA barcoding is much less laborious than laboratory rearing, allowing for analysis on many more species. In the present study we have unambiguously matched medusa and hydroid phases for 13 species. This result could be obtained only with a methodology that identifies DNA barcodes for each recognizable specimen; although Next-Generation-Sequencing of eDNA from homogenized samples might provide more data on diversity, it would not provide for matching of life-cycle stages.

**Fig 17:**
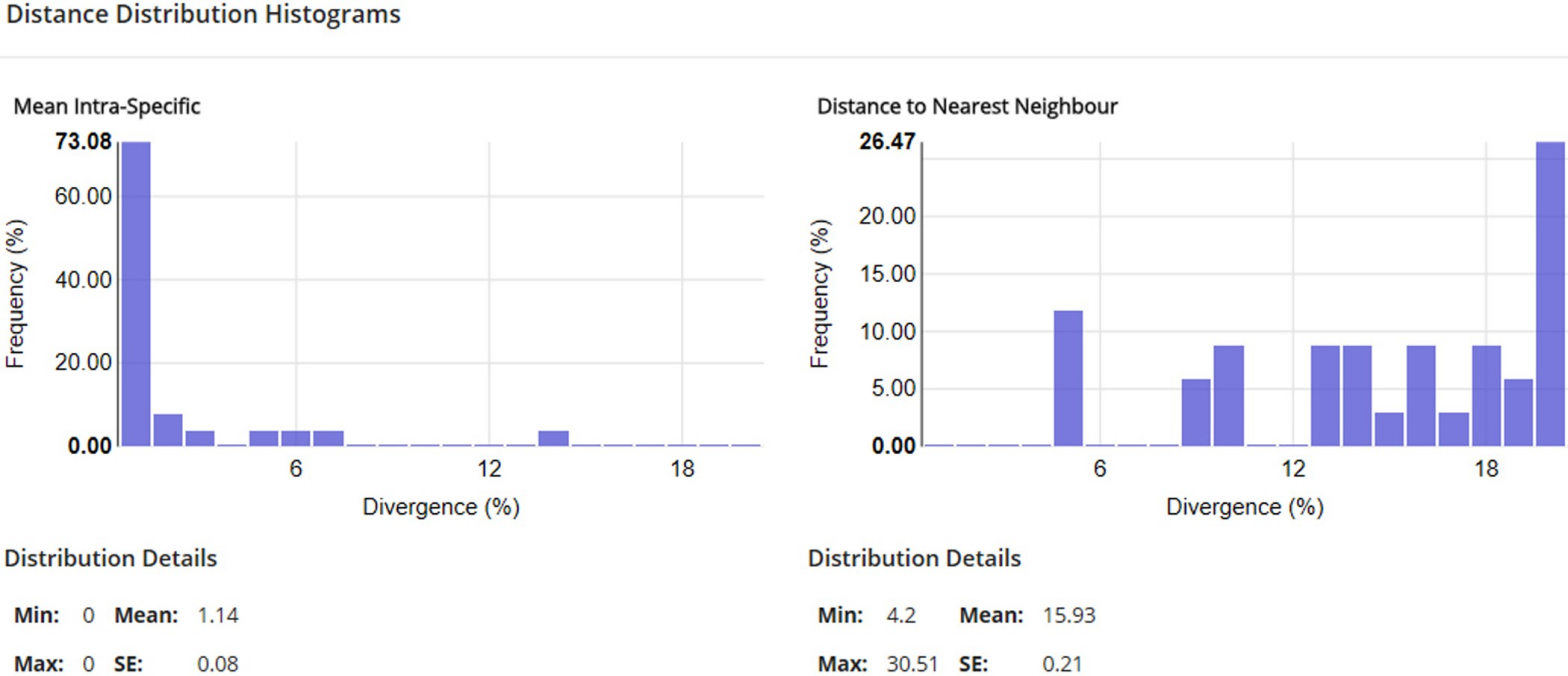
Sequence divergence in the COI Barcode for intraspecific and interspecific comparisons using the data included in this publication.

DNA barcoding has often revealed unexpected species diversity in many taxa [32], and this study leads to the same conclusion in the realm of microscopic marine animals. It shows the utility of this approach and the value of the COI Barcode for identifying cnidarian species, in spite of published arguments against its use for this phylum [12, 33, and 34]. Furthermore, our data show a clear “DNA barcode gap”; i.e. a much larger range of interspecific divergences versus intraspecific divergences in this DNA sequence for hydrozoans (Fig 17). The statement by Hebert et al, [33] was based on a collection including only 17 cnidarians, and Schuchert found that “COI of hydrozoans is often not easily amplifiable using standard primers”. Our success rate was 497 barcodes amplified from 843 specimens which, although not ideal, is a reasonably high and certainly usable success rate.
